# Sero-catalytic and Antibody Acquisition Models to Estimate Differing Malaria Transmission Intensities in Western Kenya

**DOI:** 10.1038/s41598-017-17084-9

**Published:** 2017-12-04

**Authors:** Grace E. Weber, Michael T. White, Anna Babakhanyan, Peter Odada Sumba, John Vulule, Dylan Ely, Chandy John, Evelina Angov, David Lanar, Sheetij Dutta, David L. Narum, Toshihiro Horii, Alan Cowman, James Beeson, Joseph Smith, James W. Kazura, Arlene E. Dent

**Affiliations:** 1 Center for Global Health and Diseases, Case Western Reserve University, Cleveland, OH USA; 2 Department of Molecular Medicine, Cleveland Clinic Lerner College of Medicine, Case Western Reserve University, Cleveland, OH USA; 3 Institute Pasteur, Paris, France; 4 Kenya Medical Research Institute, Kisumu, Kenya; 5 Department of Pediatrics, Riley Hospital, Indiana University, Indianapolis, IN USA; 6 Malaria Vaccine Branch, Walter Reed Army Institute of Research, Silver Spring, MD USA; 7 Laboratory of Malaria Immunology and Vaccinology, National Institutes of Allergy and Infectious Diseases, National Institutes of Health, Rockville, MD USA; 8 Department of Molecular Protozoology, Research Institute for Microbial Diseases, Osaka University, Suita, Osaka, Japan; 9 Walter and Eliza Hall Institute of Medical Research, Parkville, VIC, Australia; 10 Burnet Institute, Melbourne, Australia; 11 Center for Infectious Disease Research, Seattle, WA USA; 12 Department of Pediatrics, Rainbow Babies and Children’s Hospital, Cleveland, OH USA

## Abstract

We sought to identify a subset of *Plasmodium falciparum* antibody targets that would inform monitoring efforts needed to eliminate malaria in high transmission settings. IgG antibodies to 28 recombinant Pf antigens were measured in residents of two communities in western Kenya examined in 2003 and 2013, when the respective prevalence of asymptomatic parasitemia among children was 81 and 15 percent by microscopy. Annual seroconversion rates based on a sero-catalytic model that dichotomised antibody values to negative versus positive showed that rates were higher in 2003 than 2013 for 1 pre-erythrocytic and 7 blood-stage antigens. Antibody acquisition models that considered antibody levels as continuous variables showed that age-related antibody levels to Circumsporozoite Protein and 10 merozoite proteins increased at different rates with age in 2003 versus 2013. Both models found that antibodies to 5 proteins of the Merozoite Surface Protein 1 complex were differentially acquired between the cohorts, and that changes in antibody levels to Apical Membrane Antigen 1 suggested a decrease in transmission that occurred ~10 years before 2013. Further studies evaluating antibodies to this subset of Pf antigens as biomarkers of malaria exposure and naturally acquired immunity are warranted in endemic settings where transmission has been reduced but persists.

## Introduction

The United Nations Millennium Development Goals of 2000 inspired efforts to reduce the health burden of malaria in sub-Saharan Africa. Along with improved access to health care and artemisinin combination treatment regimens many countries instituted programs to reduce malaria transmission by expanding vector interventions such as insecticidal bed nets (ITN) and indoor residual spraying of insecticides. Remarkable decreases in the prevalence of asymptomatic *P*. *falciparum* (Pf) infection among 2–10 year old children (PfPR_2–10_), a widely used metric of transmission intensity^1,2^, and the overall incidence of uncomplicated malaria among African children were achieved by 2015^3,4^. However, recent trends in some areas of sub-Saharan Africa indicate that clinical malaria incidence may be rebounding and affecting older children, presumably as the result of slower development of clinical immunity in situations where transmission has been reduced but not ceased^5–7^. The underlying factors affecting this transition likely vary across endemic sites, *e*.*g*. differing levels of transmission before vector interventions were expanded; uneven distribution, use and replacement of ITN; insecticide resistance; changes in the dominant mosquito vector species and their biting behavior.

Areas of western Kenya bordering Lake Victoria have historically had high and relatively stable levels of Pf transmission with rapid development of naturally acquired immunity to uncomplicated malaria during childhood. Entomological studies conducted in Kisumu County during the last two decades of the 20^th^ century reported that the entomological inoculation rates were on the order of 0.65–0.82 infective bites/person/day in many communities^8,9^. Large scale epidemiological studies conducted by the US Centers for Disease Control and community surveys by ourselves and others reported that the prevalence of asymptomatic parasitemia among children ≤ 10 years was >70–80% by blood smear, and protection from uncomplicated malaria generally developed by age 3–5 years up to 2005–2006^10–14^. In 2006, the Kenya Ministry of Health greatly expanded the distribution of free long lasting ITNs^15^. ITN ownership (estimated as the proportion of households that owned at least one ITN) in a sentinel village in Kisumu County increased from ~40% in 2002 to 70% in 2010. More than 80% of households owned at least one ITN after additional distribution campaigns in 2011 and 2014^16^. Concomitant trends in the prevalence of asymptomatic parasitemia among school age children showed a reduction from ~50–60% before 2006 to ~19% in 2008 following ITN distribution, which remained stable and persisted in 2011^16,17^. Given the changing dynamics of malaria transmission, the goal of this study was to evaluate serological monitoring tools that reflect differing malaria exposures and age related acquisition of anti-malarial antibodies in settings where the level of endemicity has historically been high. Using plasma collected during a 2003 cross-sectional survey of children and adults in Kanyawegi and a 2013 cross-sectional survey in Chulaimbo, two communities in Kisumu County located 11 km from each other, we quantified IgG antibody levels to 3 pre-erythrocytic and 25 recombinant blood stage antigens and evaluated their relationship with the prevalence of asymptomatic parasitemia and age profile of uncomplicated malaria incidence in children. Cross sectional serological data have previously been analyzed by sero-catalytic models^18–21^ and, more recently, antibody acquisition/density models^22,23^. Sero-catalytic models describe the rate at which a population changes from being sero-negative to sero-positive due to malaria exposure, and how that population reverts to being sero-negative if there is a decline in transmission. Antibody acquisition models are similar to sero-catalytic models except they utilize continuous measures of antibody responses rather than binary sero-positivity status. We used both approaches to determine whether antibodies to a subset of these Pf antigens might be informative for evaluating differences in the two cohorts with respect to malaria exposure and age-related acquired immunity to uncomplicated malaria.

## Results

### Asymptomatic parasitemia and uncomplicated malaria incidence in 2003 and 2013

**Figure 1:**
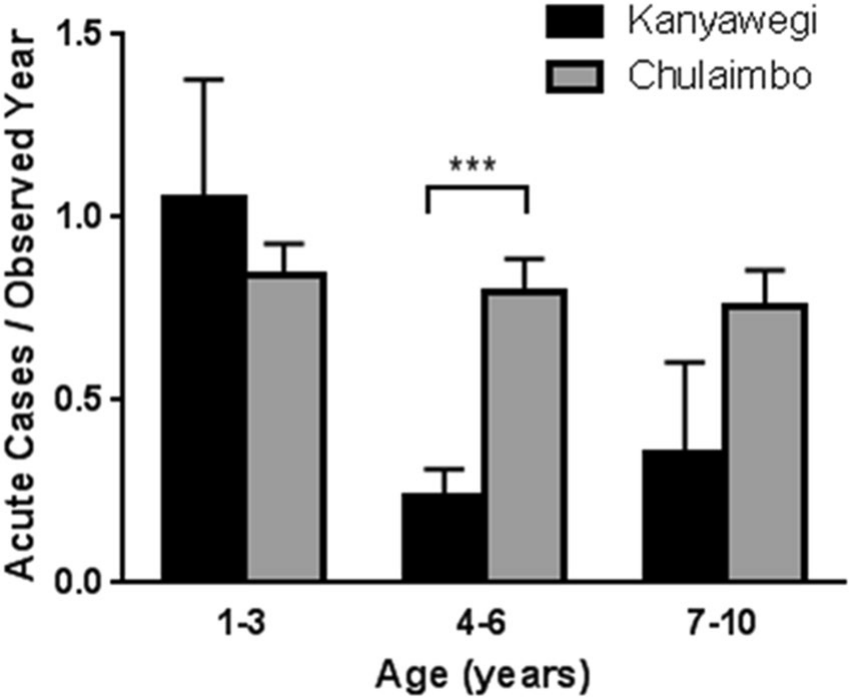
Mean number of acute uncomplicated malaria cases among children < 10 years old per observed year in the 2003 Kanyawegi and 2013 Chulaimbo cohorts with 95% CI. Statistical significance was determined within age groups by independent t-test. ***p < 0.005.

The prevalence of asymptomatic Pf infection among 1–10 year old children in Kanyawegi in 2003 was 81% (67/83) versus 15% (13/87) in Chulaimbo in 2013 (p < 0.0001); corresponding values for adults ≥ 18 years old were 32% (31/96) and 8% (4/50) (p = 0.0009). The prevalence of asymptomatic blood-stage infection among children aged 1–3, 4–6, 7–10 years was 4.1- to 7.5- fold higher in 2003 than in 2013. The age-stratified incidence of uncomplicated malaria among children from Kanyawegi and Chulaimbo is shown in Fig. 1. Malaria incidence was similar for 1–3 year olds in both cohorts (1.05 and 0.84 events per person-year). However, the incidence was significantly lower in the 2003 Kanyawegi cohort than in the 2013 Chulaimbo cohort for the 4–6 year age group (p < 0.005). The incidence in the 7–10 year age group was also lower in Kanyawegi than in Chulaimbo, but this was not statistically significant (p = 0.155). The association of age with incidence was significant for the 2003 Kanyawegi cohort (p = 0.004, one way ANOVA) but not the 2013 Chulaimbo cohort (p = 0.488). Six percent of households owned a bed net in Kanyawegi in 2003 whereas 85 percent in Chulaimbo owned a long lasting ITN in 2013.

### Age profiles of IgG antibodies in 2003 and 2013

**Figure 2:**
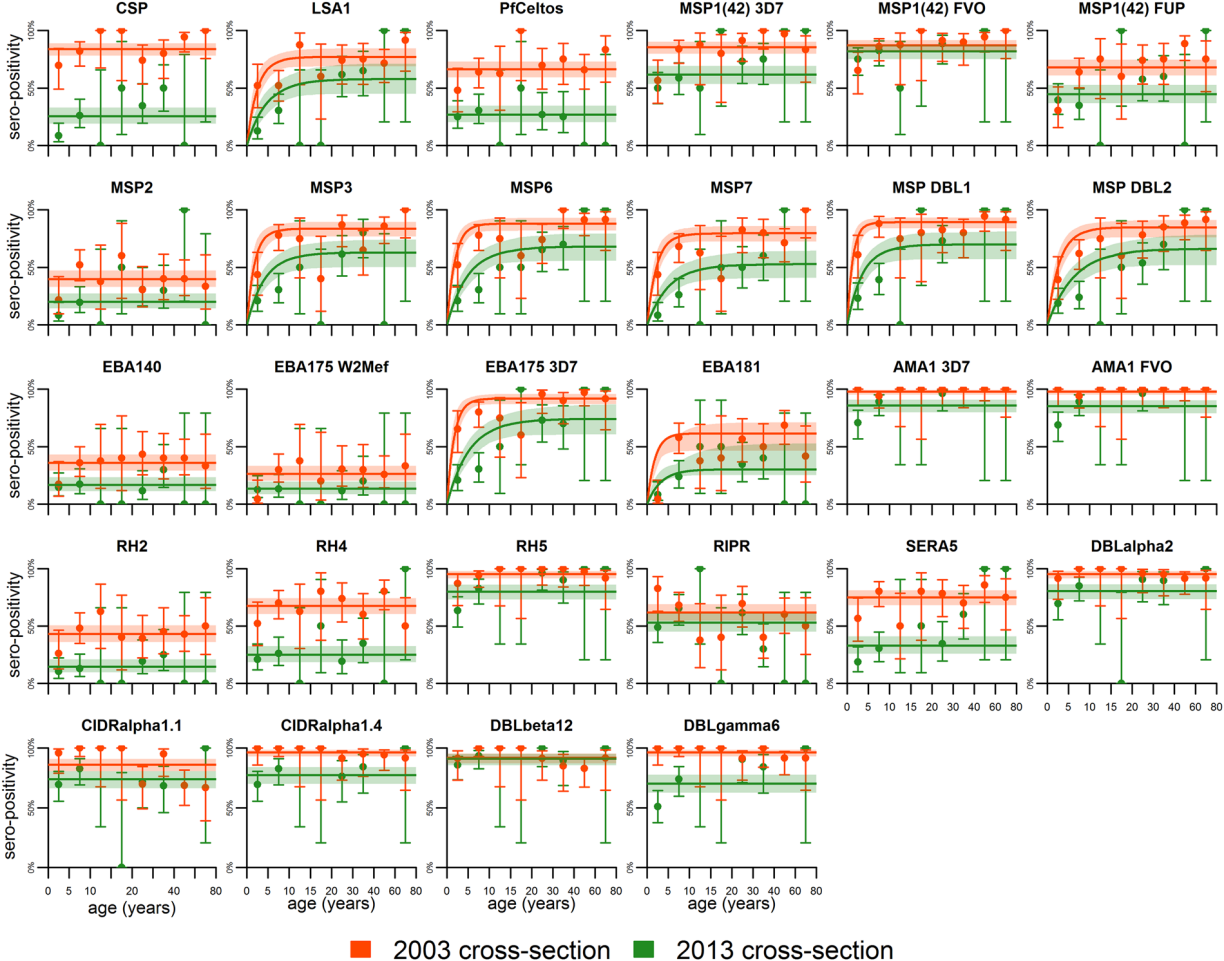
Age-related sero-positivity to 28 *Plasmodium falciparum* antigens in 2003 and 2013 cohorts. A multiplexed antibody assay was performed using recombinant antigens conjugated to microsphere beads as described previously^45^. Mean fluorescent intensity (MFI) values obtained from the assay were divided by the average MFI of malaria naïve North American controls. MFI values greater than the mean MFI + 3 standard deviations of malaria naïve controls were considered sero-positive. The data are presented as the sero-positive proportion with 95% confidence intervals in different age bins. Continuous lines represent the best model fit. The shaded areas denote the 95% credible interval of the model fit.

**Figure 3:**
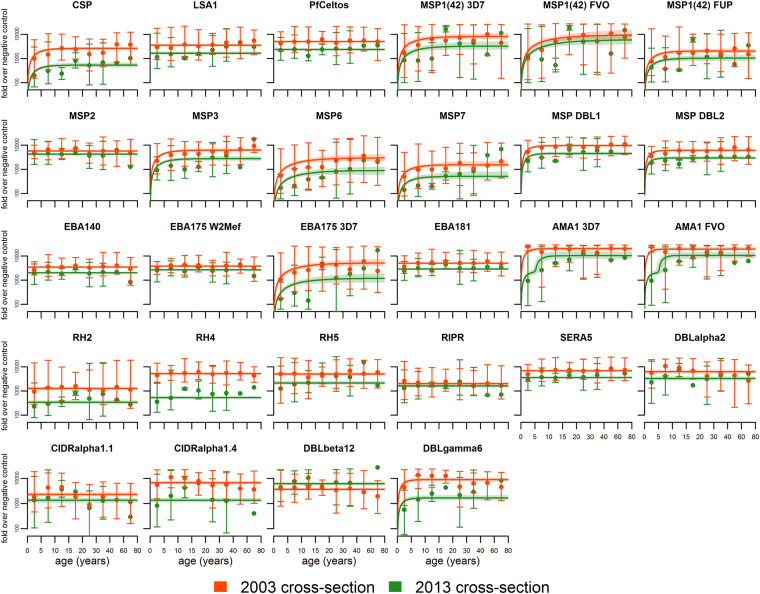
Age-related geometric mean antibody levels for 28 *Plasmodium falciparum* antigens in 2003 and 2013 antigens. A multiplexed antibody assay was performed using recombinant antigens conjugated to microsphere beads as described previously^45^. Mean fluorescent intensity (MFI) values obtained from the assay were divided by the average MFI of malaria naïve North American controls. Results are expressed as the geometric fold-increase of study participant MFI relative to the MFI of malaria naïve controls. The data are presented as geometric mean antibody levels with 95% ranges in different age bins. Continuous lines represent the best model fit. The shaded areas denote the 95% credible interval of the model fit.

Age-related sero-positivity curves for all 28 Pf proteins are presented in Fig. 2. Analogous data for the age-related changes in antibody levels, expressed as the geometric mean fold increase in antibody (mean fluorescence intensity, MFI) relative to plasma from malaria naïve adults, are shown in Fig. 3. Visual inspection of the curves in these figures suggests that there are substantially different patterns of age-related changes in sero-positivity and geometric mean antibody levels for several antigens. For example, with respect to the pre-erythrocytic antigens, sero-positivity to Circumsporozoite Protein (CSP) was relatively unchanged with increasing age in both 2003 and 2013, though the overall level of seropositivity was higher in 2003 than in 2013. In contrast, the geometric mean antibody level to CSP increased during childhood in both 2003 and 2013. With respect to Liver Stage Antigen 1 (LSA1), there was an age-related progressive increase in sero-positivity up to age ~10 years in both 2003 and 2013, with a more rapid rise in 2003 than in 2013 (Fig. 2), while there was no discernable change in age-related mean antibody levels to LSA1 in either 2003 or 2013 (Fig. 3). Discordance in the visual patterns and/or levels for age-related sero-positivity and geometric mean antibody levels to several merozoite antigens were also evident. These included the 3 allelic variants of the C-terminal 42 kDa fragment of Merozoite Surface Protein 1 (MSP1(42)) we examined, MSP2, Erythroycte Binding Antigen 140 (EBA140), both alleles of EBA175, EBA181, both alleles of Apical Membrane Antigen 1 (AMA1), Reticulocyte Binding-like Homologous protein 2 (RH2), RH4 and Serine Rich Antigen 5 (SERA5). The patterns of age-related sero-positivity and geometric mean antibody levels to proteins corresponding to the cysteine-rich interdomain regions (CIDR) subtypes α1.1 and α1.4, which form part of Domain Casettes 8 and 13 of Pf Erythrocyte Membrane Protein 1 (PfEMP1), were similar, with high levels in all age groups in both 2003 and 2013. This complexity of age-related patterns and levels underscores the importance of modeling in analyzing sero-positivity and antibody magnitude data obtained from cross-sectional malaria surveys.

### Analyses using sero-catalytic and antibody acquisition models

The age and exposure dependent changes in sero-prevalence and antibody levels were analysed using sero-catalytic models as described by Drakeley *et al*.^19^, and antibody acquisition models as described by Yman *et al*.^22^. Three variants of each of these models were tested. Model 0 assumed no age-dependent variation in sero-prevalence or antibody levels. Model 1 assumed a constant rate of sero-conversion or antibody acquisition in both cross-sections. Model 2 was similar to Model 1 except allowed for inclusion of a prior historical reduction in transmission in the 2013 cross-section.

Results for the best fitting model for each of the antigens according to the sero-catalytic and antibody acquisition models are presented in Tables 1 and 2, respectively. For the sero-catalytic model, no difference in age-related seroconversion rate (SCR) for the 2003 cross- section versus 2013 cross-section was observed for 20 proteins (best fitting Model 0). Note that comparison of changes in the annual SCR for the 2003 and 2013 cohorts could not be made for several antigens because of apparently high immunogenicity whereby nearly 100% of children ≤ 10 years old were sero-positive in 2003 or in both 2003 and 2013, *e*.*g*. Circumsporozoite Protein (CSP), three allelic variants of MSP1(42), two allelic variants of AMA1, RH5, PfEMP1 Duffy binding-like domain alpha 2 (DBLα2), CIDRα1.1, CIDRα1.4, DBLβ12 and DBL$\gamma$6. Other Pf antigens showed relatively lower immunogenicity such that seroprevalence in adults was ≤50% in either or both 2003 and 2013, *e*.*g*. Pf Cell-Traversal Protein for Ookinetes and Sporozoites (PfCeltos), MSP2, EBA175 W2Mef, RH2 and RH4. The data from 8 of 28 antigens – LSA1, MSP3, MSP6, MSP7, MSPDBL1, MSPDBL2, EBA175 3D7, EBA181 – favored a model fit with a constant SCR (denoted λ in Table 1). Model 2 was not selected for any antigen.

**Table 1:** Sero-catalytic model parameters. Results shown are the median and 95% credible intervals from the posterior parameter distribution estimated using Bayesian Markov Chain Monte Carlo (MCMC) methods.

Antigen	Model	*λ*_03_ or *P*_03_	*λ*_13_ or *P*_13_	*ρ* (year^−1^)	*t*_half_ (year)
CSP	0	84% (78%, 89%)	26% (19%, 33%)		
LSA1	1	0.155 (0.103, 0.247)	0.063 (0.043, 0.096)	0.046 (0.028, 0.093)	15.1 (7.5, 24.8)
PfCeltos	0	66% (59%, 73%)	27% (20%, 34%)		
MSP1(42) 3D7	0	86% (80%, 90%)	62% (54%, 69%)		
MSP1(42) FVO	0	87% (82%, 92%)	82% (75%, 88%)		
MSP1(42) FUP	0	68% (61%, 75%)	45% (37%, 53%)		
MSP2	0	40% (33%, 47%)	20% (14%, 27%)		
MSP3	1	0233 (0.158, 0.362)	0.076 (0.053, 0.114)	0.046 (0.023, 0.088)	15.1 (7.9, 30.1)
MSP6	1	0.242 (0.166, 0.371)	0.071 (0.049, 0.103)	0.032 (0.015, 0.066)	21.7 (10.5, 46.2)
MSP7	1	0.185 (0.123, 0.300)	0.052 (0.034, 0.081)	0.048 (0.024, 0.098)	14.4 (7.1, 28.9)
MSPDBL1	1	0.347 (0.231, 0.571)	0.096 (0.067, 0.142)	0.041 (0.020, 0.084)	16.9 (8.3, 34.7)
MSPDBL2	1	0.155 (0.106, 0.235)	0.053 (0.037, 0.079)	0.028 (0.012, 0.058)	24.8 (12.0, 57.8)
EBA140	0	36% (29%, 43%)	17% (11%, 23%)		
EBA175 W2Mef	0	26% (20%, 33%)	13% (9%, 20%)		
EBA175 3D7	1	0.278 (0.192, 0.437)	0.071 (0.049, 0.102)	0.024 (0.009, 0.053)	28.9 (13.1, 77.0)
EBA181	1	0.131 (0.082, 0.252)	0.052 (0.028, 0.096)	0.097 (0.037, 0.222)	7.1 (3.1, 18.7)
AMA1 3D7	0	98% (95%, 99%)	86% (80%, 91%)		
AMA1 FVO	0	98% (95%, 99%)	85% (79%, 90%)		
RH2	0	43% (36%, 51%)	15% (10%, 21%)		
RH4	0	68% (60%, 74%)	25% (18%, 32%)		
RH5	0	95% (91%, 98%)	80% (73%, 86%)		
RIPR	0	62% (55%, 69%)	53% (45%, 61%)		
SERA5	0	75% (68%, 81%)	33% (26%, 41%)		
DBLα2	0	95% (91%, 98%)	80% (73%, 86%)		
CIDRα1.1	0	86% (80%, 9%)	74% (66%, 81%)		
CIDRα1.4	0	96% (93%, 98%)	77% (70%, 84%)		
DBLβ12	0	92% (87%, 95%)	91% (86%, 95%)		
DBLγ6	0	96% (93%, 98%)	70% (62%, 77%)		

All parameters were assumed to have improper uniform priors. Of the three models tested, the best fit was selected using the Deviance Information Criterion (DIC). In the case where Model 0 was the best fit, the proportion seropositive *P*_*i*_ is presented. In the case where Model 1 was the best fit, the seroconversion rate *λ*_*i*_ is presented. Model 2 was not selected as the best fit for any of the antigens. Annual seroreversion rate is indicated by the symbol *ρ* and *t*_half_ (year) indicates the half-life of sero-reversion: the time taken for 50% of a population of sero-positive individuals to revert to being sero-negative in the absence of ongoing transmission.

**Table 2:** Antibody acquisition model parameters. Results shown are the median and 95% credible intervals from the posterior parameter distribution estimated using Bayesian Markov Chain Monte Carlo (MCMC) methods.

Antigen	Model	*α*_03_	*α*_13_	*γ*_13_	*τ*_*c*,13_ (year)	*r* (year^−1^)	*t*_half_ (year)	*σ*
CSP	1	360 (273, 505)	75 (59, 100)			0.14 (0.09, 0.22)	5.0 (3.2, 7.4)	0.95 (0.89, 1.03)
LSA1	0	3486 (3078, 3949)	1634 (1413, 1889)					0.89 (0.83, 0.96)
PfCeltos	0	4935 (4498, 5467)	2335 (2088, 2610)					0.69 (0.64, 0.75)
MSP1(42) 3D7	1	661 (466, 980)	270 (197, 378)			0.08 (0.05, 0.14)	8.4 (4.8, 15.2)	1.46 (1.35, 1.58)
MSP1(42) FVO	1	447 (310, 663)	277 (200, 392)			0.05 (0.02, 0.10)	14.3 (7.3, 38.2)	1.63 (1.51, 1.76)
MSP1(42) FUP	1	277 (199, 437)	138 (104, 204)			0.14 (0.09, 0.25)	4.9 (2.7, 7.8)	1.10 (1.02, 1.19)
MSP2	0	5671 (5204, 6180)	4202 (3805, 4634)					0.61 (0.57, 0.66)
MSP3	1	767 (610, 991)	346 (283, 430)			0.12 (0.09, 0.17)	5.7 (4.1, 7.7)	0.81 (0.76, 0.88)
MSP6	1	158 (119, 211)	49 (38, 63)			0.05 (0.02, 0.08)	14.3 (8.8, 29.4)	1.27 (1.17, 1.37)
MSP7	1	155 (116, 213)	54 (42, 71)			0.09 (0.06, 0.14)	7.5 (4.8, 11.8)	1.15 (1.06, 1.24)
MSPDBL1	1	2151 (1703, 2861)	1017 (832, 1295)			0.23 (0.17, 0.31)	3.1 (2.2, 4.1)	0.63 (0.59, 0.68)
MSPDBL2	1	1768 (1302, 2805)	858 (656, 1284)			0.29 (0.20, 0.49)	2.4 (1.4, 3.4)	0.65 (0.60, 0.70)
EBA140	0	3439 (3178, 3725)	2024 (1847, 2218)					0.56 (0.52, 0.61)
EBA175 W2Mef	0	3801 (3546, 4078)	2651 (2446, 2881)					0.50 (0.46, 0.54)
EBA175 3D7	1	302 (224, 413)	68 (52, 91)			0.06 (0.03, 0.09)	12.3 (7.6, 22.4)	1.37 (1.28, 1.48)
EBA181	0	5023 (4648, 5440)	2870 (2621, 3139)					0.56 (0.52, 0.61)
AMA1 3D7	2	5616 (3983, 7374)	64308 (16442, 98032)	1.0% (0.6%, 3.4%)	10.6 (9.0, 16.0)	0.29 (0.20, 0.36)	2.4 (1.9, 3.5)	1.22 (1.14, 1.32)
AMA1 FVO	2	5755 (4244, 7496)	66782 (20315, 96709)	1.0% (0.6%, 2.9%)	10.5 (8.2, 16.1)	0.30 (0.22, 0.38)	2.3 (1.7, 3.8)	1.21 (1.13, 1.32)
RH2	0	1260 (1092, 1452)	342 (290, 404)					1.03 (0.96, 1.12)
RH4	0	5250 (4695, 5881)	533 (469, 608)					0.80 (0.75, 0.87)
RH5	0	5016 (4347, 5796)	2155 (1815, 2557)					1.04 (0.97, 1.12)
RIPR	0	2004 (1770, 2265)	1608 (1390, 1854)					0.88 (0.82, 0.95)
SERA5	0	6878 (6295, 7494)	3583 (3244, 3957)					0.62 (0.58, 0.67)
DBLα2	0	6275 (5379, 7337)	3264 (2697, 3939)					1.10 (1.02, 1.20)
CIDRα1.1	0	2297 (1914, 2747)	1337 (1072, 1668)					1.25 (1.16, 1.35)
CIDRα1.4	0	6679 (5762, 7776)	1351 (1072, 1668)					1.07 (0.99, 1.16)
DBLβ12	0	3661 (3215, 4158)	6092 (5243, 7114)					0.91 (0.85, 0.99)
DBLγ6	1	2334 (1677, 3547)	437 (323, 634)			0.26 (0.18, 0.41)	2.7 (1.7, 3.9)	1.07 (0.99, 1.16)

All parameters were assumed to have improper uniform priors. Of the three models tested, the best fit was selected using the Deviance Information Criterion (DIC). In the case where Model 0 was the best fit, *α*_*i*_ is the geometric mean titre (GMT). In the cases where Models 1 or 2 are the best fit, *α*_*i*_ is the antibody acquisition rate. *r* is the antibody decay rate, and *t*_*half*_ is the antibody half-life – the time taken for antibody levels to reduce by 50% in the absence of exposure. *τ*_*c*,13_ is the time of reduction of transmission in the 2013 cohort, where γ_13_ is the ratio of transmission after the reduction compared to before reduction.

For the antibody acquisition models (Table 2), we observed that the geometric mean antibody levels to 15 of the 28 antigens did not change according to age in 2003 or 2013 (best fitting Model 0). This included 2 of 3 pre-erythroyctic proteins (LSA1 and PfCeltos), 8 merozoite proteins that included all of the Pf RH proteins, MSP2, MSP3, EBA140, EBA175 W2Mef, EBA181, and 4 of the 5 DBL and CIDR domains corresponding to the extracellular head of PfEMP1 Domain Cassettes 8 and 13 thought to contribute to cerebral malaria pathogenesis^24,25^. Geometric mean antibody levels to 11 proteins revealed an age related increase that differed in the 2003 and 2013 cohorts, e.g. three alleles of MSP1(42), MSP6, MSP7, MSPDBL1, MSPDBL2, EBA175 3D7, SERA5, and PfEMP1 DBLγ6 (model 1). With respect to AMA1, the best fit of the data was consistent with model 2. Notably, there was an increase in geometric mean antibodies to both AMA1 3D7 and FVO over age 10.6 years (95% CI 9.0, 16.0) and 10.5 years (95% CI 8.2, 16.1) years, respectively. However, this change was not seen in the sero-prevalence curves (Fig. 2) due to the very high degree of immunogenicity of AMA1, as reported previously^19,26^.

**Figure 4:**
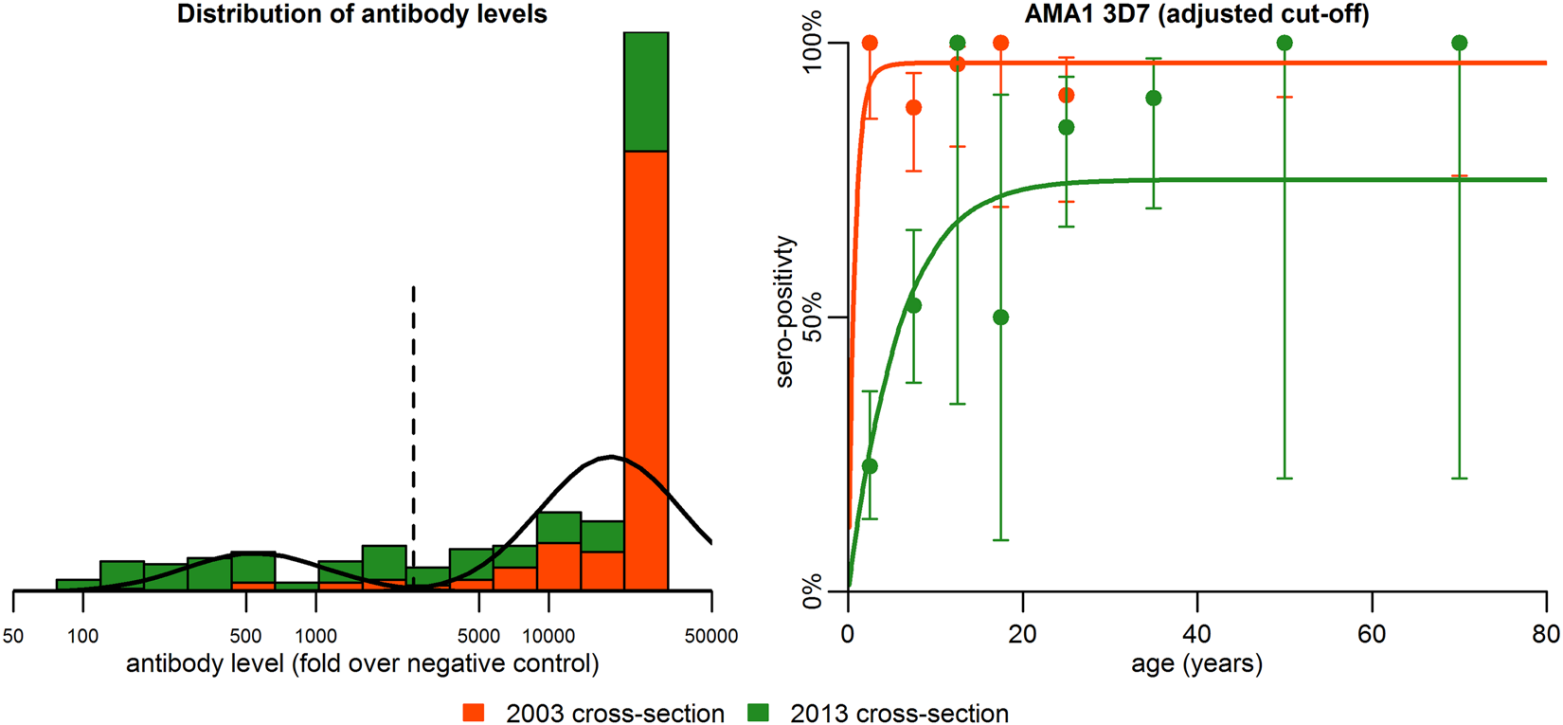
Seropositivity to Apical Membrane Antigen-1 3D7 allelic variant following upward adjustment of cut-off for seropositivity. The left panel shows that the cut-off for seropositivity was adjusted by fitting a mixture of two Normal distributions (solid line) to the data on antibody levels, moving from 164-fold greater than malaria naïve plasma as in Fig. 2 to 2,600-fold greater than malaria naïve plasma (indicated by the dashed vertical line). The best fit model to the revised data are shown in the right panel.

To explore the issue of high immunogenicity of AMA1 in more detail, we chose to reexamine the sero-prevalence data using a higher cut-off value relative to malaria naïve plasma. The original selection of cut-off for AMA1 3D7 sero-positivity based on the mean + 3 standard deviations relative to malaria naïve controls (equal to 164-fold greater than negative controls) resulted in 98% of individuals being sero-positive in the 2003 cohort, and 86% sero-positive in the 2013 cohort. This high degree of sero-positivity obscures the information on past trends in transmission intensity that can be seen by looking at the continuous measurements of antibody levels. We therefore selected an alternative choice of cut-off by fitting a mixture of two Normal distributions to the log antibody level. The results of this adjustment are presented in Fig. 4. As revealed by the less rapid increase in sero-positivity under age 10 years in the 2013 compared with the 2003 cohort, by selecting an optimal cut-off we regained the information on historical transmission patterns.

### Correlations between antibody responses to various antigens

**Figure 5:**
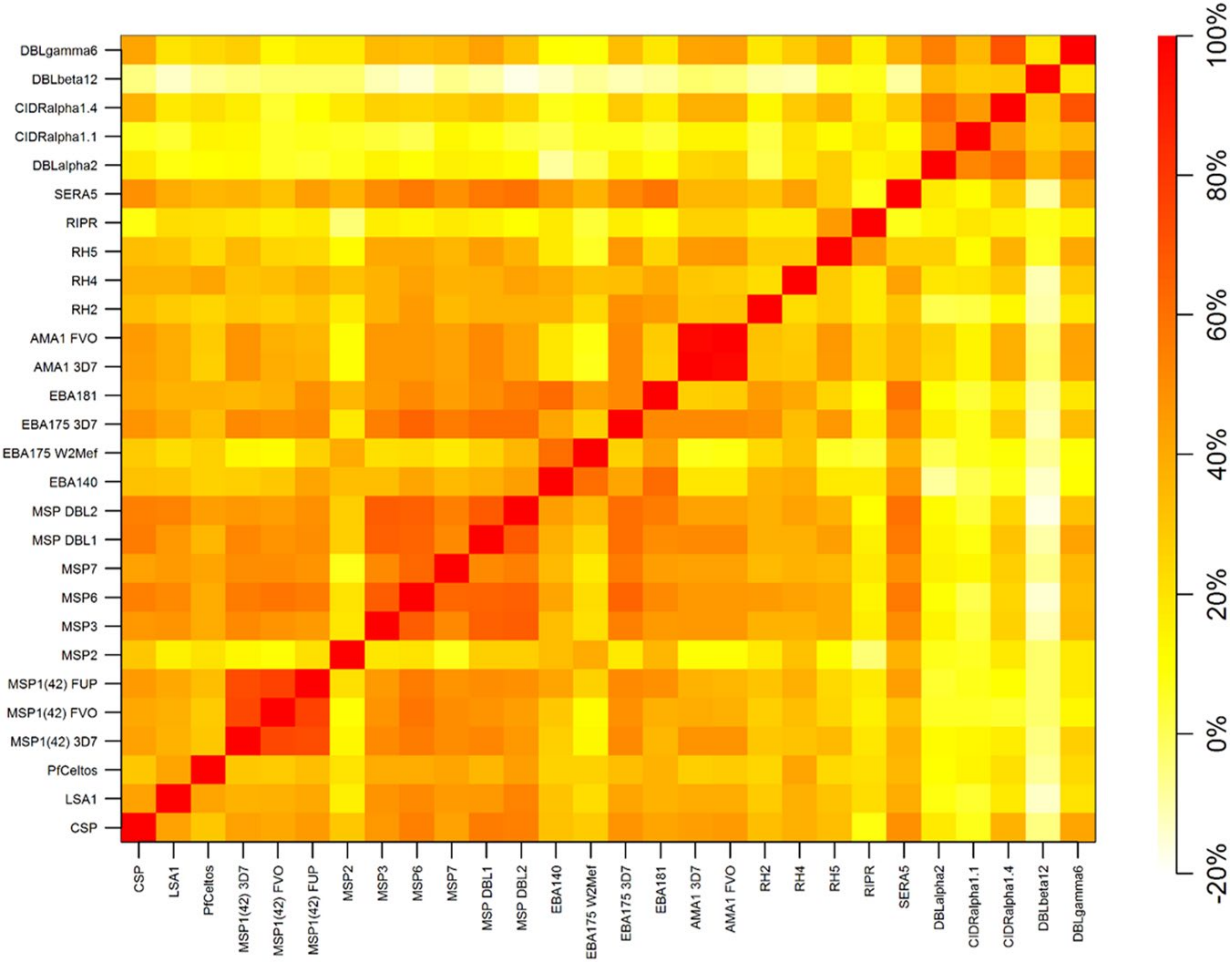
Heat map of correlation of antibody levels for two-way comparisons of 28 *Plasmodium falciparum* antigens. Increasing intensity of yellow to orange to red coloring correlates with an increasing degree of correlation between antibody levels.

We also examined the correlation between antibody levels to pre-erythrocytic, merozoite, PfEMP1 and allelic variants of selected merozoite proteins for which we tested more than one allele. Figure 5 is a heat map illustrating these correlations for every combination of two antigens. Supplementary Data provides the precise level of correlation, ranging from nil to 1. Antibodies corresponding to PfEMP1 DBL and CIDR domains showed 29 to 62% correlation with each other (orange to light red coloring in Fig. 5), with less correlation with pre-erythrocytic or merozoite proteins (yellow to light orange coloring). Antibody levels to peripheral proteins that are not anchored to the merozoite surface membrane by glycophosphatidylinositol anchors and form MSP1 complexes which mediate adherence to the erythrocyte prior to invasion (MSP3, MSP6, MSP7, MSPDBL1 and MSPDBL2) showed a modest to strong correlation with each other^27^. The strongest correlation (~69%) was for MSP3-MSP6, MSP3-MSPDBL1, MSP3-MSPDBL2, and MSPDBL1-MSPDBL2, with slightly weaker correlations with antibody levels to MSP7 (52 to 64% for the other peripheral proteins). With respect to antibody reactivity to variant alleles of the same antigen, antibody levels to AMA1 3D7 and FVO and MSP1(42) 3D7, FVO and FUP correlated by > 90% and > 70%, respectively. Antibody levels to EBA175 W2Mef and 3D7 correlated by only 28%.

### Breadth of antibody responses based on sero-positivity

The breadth of Pf antigens identified by IgG antibodies was calculated by counting the number of antigens from a total of 28 for which a study participant in the 1–3, 4–6, 7–10, and ≥18 year age groups was seropositive. Breadth increased with age in both the 2003 and 2013 cohorts (Supplementary Fig. S1). The 2003 cohort had median values of sero-positivity for 20, 18, 24, and 23 of the Pf antigens for the four age groups described above. The same age groups in the 2013 cohort were antibody-positive for 10, 12, 13, and 18 antigens, respectively (p < 0.0001–0.05 for 2003 vs. 2013).

### Antibody levels in children with positive versus negative blood smears

In order to determine whether asymptomatic infection affected antibody levels, we compared antibody levels of asymptomatic blood smear positive children to asymptomatic blood smear negative children in both cohorts. No significant difference in seropositive prevalence or geometric mean antibody levels to any of the antigens was found.

## Discussion

A goal of this and other studies of malaria serology is to improve population level diagnostics that can be used to inform malaria control and elimination efforts in endemic settings. Extracts of blood stage parasites, immunofluorescent assays of infected erythrocytes, recombinant proteins and microarrays displaying thousands of Pf protein features based on the 3D7 genome sequence have been used to screen and validate serologic correlates of Pf exposure and incident cases of clinical malaria^18,28–34^. In this study, we used a panel of 28 recombinant Pf proteins and compared two analytic approaches that have been used to analyze data from cross-sectional serological surveys to evaluate the impact of differing transmission dynamics on Pf antibody responses. Our multiplexed antibody assay included Pf antigens that have previously been evaluated as serologic tools, *e*.*g*. CSP, MSP1, MSP2, AMA1 and EBA175 as well as those for which there is less information regarding their potential relevance to monitoring transmission intensity and age-related acquired immunity to symptomatic malaria, *e*.*g*. LSA1, PfCeltos, PfRH proteins, EBA181.

Taking into consideration the strengths and weaknesses of the two analytic approaches to evaluate serologic data from cross-sectional surveys^22^, our results suggest it may be reasonable to select for further validation antibody responses to a subset of antigens for which there was a difference in age related seroconversion and antibody levels in both 2003 and 2013, when the prevalence of asymptomatic parasitemia in children was 81 and 15 percent, respectively. Antigens meeting this criteria included MSP3, MSP6, MSP7, MSPDBL1, MSPDBL2 and EBA175 3D7 (Figs 2 and 3, Tables 1 and 2). All but one of these antigens (EBA175) are part of the MSP1 complex with peripheral merozoite surface proteins (MSP3, MSP4, MSP6, MSP7, MSPDBL1 and MSPDBL2) that is functionally essential to the initial, low affinity attachment of merozoites to the erythrocyte surface^27^. MSP1(42), the only one of several proteolytically processed fragment of MSP1 we examined^35,36^, did not prove informative in the sero-catalytic model but did so in the antibody acquisition model, largely because of its high immunogencity due to its greater abundance relative to other MSPs. Further, processed fragments of MSP1 (along with the other MSPs) are shed into the blood prior to erythrocyte invasion while the merozoite membrane-anchored MSP1(19) fragment is carried into the erythrocyte, thereby enhancing exposure of the shed MSP polypeptides to the immune system. Notably, examination of the correlation between levels of antibodies to proteins within the MSP1 complex showed a strong correlation among several pair-wise comparisons. For example, there was 69% correlation between antibody levels to MSP3 and MSP6, MSP3 and MSPDBL1, MSP3 and MSPDBL2, MSP6 and MSPDBL1, MSP6 and MSPDBL2, and ~60% between MSPDBL1 and MSPDBL2. These correlations were stronger than those observed for MSP7 with any of the former proteins or the 3 variant alleles of MSP1(42) we examined (Fig. 4). It remains to be determined whether these serologic observations correlate with functional activity, *e*.*g*. does plasma from high antibody responders to the various proteins within the MSP1 complex have a greater ability to impair erythrocyte invasion or opsonic phagocytosis of merozoites relative to low responders, and is this functional activity predictive of protection from symptomatic malaria^37^.

A second major finding from our results relates to how information from the antibody acquisition model can be leveraged to adjust the parameters of the sero-catalytic model in a manner that can overcome the problem of high immunogenicity of many blood stage antigens. Many of the proteins we examined had high immunogenicity: the proteins corresponding to the extracellular head structure of PfEMP1 and MSP1(42) FVO had sero-prevalence during childhood of nearly 100% in 2003. When the standard sero-catalytic model cut off for sero-positivity to both allelic variants of AMA1 was set equal to the mean plus 3 standard deviations of malaria naïve plasma, AMA1 dropped out as a candidate for further evaluation. However, after taking into consideration that the antibody acquisition model revealed that the AMA1 data best fit a reduction in transmission ~10 years before the 2013 cross-section was performed (Model 2 was the best fit in Table 2), we adjusted the cut off for positivity to a higher level in the sero-catalytic model and produced seroprevalence curves that revealed significant differences in the seroconversion rates for 2003 compared to 2013. Our findings therefore confirm those from other studies that antibodies to AMA1 are highly effective for sero-surveillance^21,22^, but subject to an appropriate choice of cut-off. Further studies with AMA1 and other highly immunogenic Pf antigens would be of particular relevance to transmission dynamics in western Kenya. Wong *et al*.^26^ evaluated annual parasite prevalence and associated changes in antibody prevalence, magnitude and seroconversion rates to AMA1, the 19 kD C-terminus of MSP1 and CSP in 5 cohorts of 1–5 year children residing in the Asembo Bay area from 1994 to 2009. A large community trial of insecticidal nets was conducted in this area from 1997 to 1999^38^, and bed net distribution was expanded to antenatal clinics in 2004 and children < 5 years in 2006. Wong *et al*. observed that reductions in the prevalence of AMA1 sero-positivity and levels were most informative with respect to their correlation with decreases in the annual parasite prevalence from 1994 to 2007, followed by an upward trend in 2008 and 2009. The authors concluded that studies examining antibody responses to additional Pf antigens and other exposure related variables, such as the seroreversion rate, may be informative for monitoring the impact of vector interventions on serologic correlates of exposure in high endemicity regions such as western Kenya where transmission intensity has decreased but persists at moderate levels. Tracking antibodies to immunodominant merozoite antigens such as AMA1 may also be useful to identify residual areas of transmission where intense vector and other malaria interventions have been reported to have a profound impact such as was observed in Bioko Island, Equatorial Guinea^29^.

Additional observations regarding the broadening of the antigenic breadth of antibodies to various Pf antigens with increasing age among children, and more rapid development of this antibody repertoire that correlates positively with the prevalence of asymptomatic childhood parasitemia (Supplementary Fig. S1) confirms previous reports that panels of Pf antigens presented in formats such as ELISA, multiplexed recombinant proteins and protein microarrays generated by cell-free *E*. *coli* expression systems. These studies have reported that the breadth of antibody responses increases with age in high transmission areas such as western Kenya^12^, the breadth and intensity of antibodies are greatest when measured in proximity to a recent infection^32^ and antibody breadth is best appreciated by examining not only well characterized Pf proteins that have been selected on the basis on their high immunogenicity or relevance to vaccine development, but also novel and less well characterized proteins for which this information is not yet available^39^.

Finally, it is important to note that our observations will require validation in additional study populations characterized in more depth. Limitations to our study design include the lack of complementary entomological data, prospective data describing changes in the prevalence of asymptomatic parasitemia and malaria serology over time in the same community, and limited population size that does not have sufficient power to detect changes in the incidence of severe malaria. Furthermore, the models analyze data from one antigen at a time. It may be possible to extract additional information on previous exposure by utilizing data from multiple antigens and accounting for the correlation structure. Such studies will ideally be done in circumstances where they may have the greatest relevance to ongoing malaria dynamics in sub-Saharan Africa, particularly in regions where rebounds in malaria transmission and morbidity are suspected^40–43^.

## Methods

### Ethical approval for human studies

Observational studies of the study participants from Kenya were approved by the Institutional Review Board at University Hospitals Cleveland Medical Center and the Kenya Medical Research Institute Ethical Review Committee. Study participants ≥ 18 years old gave written informed consent. Parents/guardians gave written informed consent for child participants. All experiments were performed in accordance with guidelines and regulations for human research subject protections.

### Study sites and participants

The 2003 cohort examined for IgG antibodies to Pf antigens and determination of asymptomatic parasitemia included 97 healthy asymptomatic 1–14 year old children and 95 adults ≥ 18 years old residing in the Kanyawegi sub-location of Kisumu County (previously referred to as Nyanza Province)^13,14^. Plasma samples from all study participants were examined. The 2013 cohort included 97 healthy asymptomatic 2–10 year old children and 50 adults residing in the 10 km radius region served by the Chulaimbo health center. Plasma samples utilized were a subset of a larger study of 400 children and 100 adults described below. Selection of this subset included all available samples collected from healthy children and adults in 2013 that were in hand at the start of testing. The distance from Kanyawegi to Chulaimbo is ~11 km.

### Malaria incidence and asymptomatic parasitemia

The annual incidence of uncomplicated malaria (defined by a recent history of malaria symptoms, axillary temperature ≥ 37.5 °C, Pf positive blood smear followed by administration of antimalarial drugs) is expressed as the number of uncomplicated malaria events per person-year of observation, calculated as described by Bloland and coworkers^11^. Clinical data from Kanyawegi residents were calculated from 12 months of active and passive malaria surveillance that was collected in 2002–2003^44^. Data from Chulaimbo residents were calculated from 24 months of bi-annual active surveillance and continual passive surveillance of 400 1–10 year old children and 100 adults from July 2013 through July 2015. Estimates of bed net use were based on responses to a questionnaire that asked if an adult or child of a parent/guardian occupied a household that owned at least one bed net.

### Collection of blood and plasma for antibody studies

Peripheral blood was collected from study participants by venipuncture, plasma isolated, and frozen at −80 °C in July 2003 (Kanyawegi) and in 2013 (Chulaimbo). Thick and thin blood smears were stained with Giemsa to determine asymptomatic parasitemia. Microscopic inspection of blood smears involved examination of visual fields that included at least 200 leukocytes. Parasite density was calculating assuming 8000 leucocytes/μL blood.

### Measurement of IgG antibodies to recombinant *Plasmodium falciparum* proteins

Salient references to Pf antigens and laboratories providing the 28 recombinant proteins we used to measure IgG antibodies are described in Supplementary Table S1. Proteins were conjugated to MAGPIX (Luminex) and non-magnetic microsphere bioplex beads (Bio-Rad) according to the manufacturer’s instructions with slight modifications^45^. Briefly, varying concentrations of each protein were coupled to beads in order to obtain an optimal antibody signal determined by testing plasma pooled from 20 healthy adult residents of Kanyawegi bled in 2000. The plasma pool was diluted serially and the linear range of antibody binding determined from the standard curve for each bead. Plasma was incubated with a master mix of Pf protein conjugated beads at a 1:1 ratio for final dilutions of 1:100 and 1:1000. Antibody magnitude was quantified using the dilution within the linear range of the positive control pool. The secondary antibody was R-Phycoerythrin AffiniPure F(ab’)_2_ fragment goat anti-human IgG F(ab’)_2_ (Jackson ImmunoResearch Laboratories). Mean fluorescent intensity (MFI) values obtained from the assay were divided by the average MFI of malaria naïve North American controls on the same plate. Results are expressed as the fold-increase of study participant MFI relative to the MFI of malaria naïve controls. MFI values greater than the mean MFI + 3 standard deviations of malaria naïve controls were considered positive. MFI values below this value were set to 1, which is equivalent to malaria naïve controls.

### Sero-catalytic and antibody acquisition models

Age-dependent variation in sero-prevalence was modelled using sero-catalytic models^20^, and age-dependent variation in antibody levels was modelled using antibody acquisition models^22^. Three variants of these model were fitted reflecting different assumptions on antigen immunogenicity and historical malaria exposure were tested.

Model 0: no age-dependent variation in sero-positivity, with sero-prevalence in the 2003 cross-section equal to *P*_03_, and sero-prevalence in 2013 equal to *P*_13_.

Model 1: sero-positivity increases at a constant rate with age such that λ signifies annual seroconversion rate (SCR) and sero-prevalence at age *a* is given by:

i $$\begin{array}{c}{P}_{03}(a)=\frac{{\lambda }_{03}}{{\lambda }_{03}+\rho }(1-{e}^{-({\lambda }_{03}+\rho )a})\\ {P}_{13}(a)=\frac{{\lambda }_{13}}{{\lambda }_{13}+\rho }(1-{e}^{-({\lambda }_{13}+\rho )a})\end{array}$$

Model 2: sero-positivity increases constantly with age, but for the samples taken in the 2013 cross-section, we account for a sharp drop in transmission *T*_*c*_ years ago. The sero-prevalence at age *a* is given by:

ii $$\begin{array}{l}{P}_{03}(a)=\tfrac{{\lambda }_{03}}{{\lambda }_{03}+\rho }(1-{e}^{-({\lambda }_{03}+\rho )a})\\ {P}_{13}(a)=\{\begin{array}{cc}\tfrac{{\lambda }_{13,c}}{{\lambda }_{13,c}+\rho }(1-{e}^{-({\lambda }_{13,c}+\rho )a}) & a\le {T}_{c}\\ \tfrac{{\lambda }_{13,c}}{{\lambda }_{13,c}+\rho }+\tfrac{({\lambda }_{13,0}-{\lambda }_{13,c})}{({\lambda }_{13,0}+\rho )({\lambda }_{13,c}+\rho )}{e}^{-({\lambda }_{13,c}+\rho )(a-{T}_{c})}-\tfrac{{\lambda }_{13,0}}{{\lambda }_{13,0}+\rho }{e}^{-({\lambda }_{13,c}+\rho )a}{e}^{-({\lambda }_{13,0}-{\lambda }_{13,c}){T}_{c}} & a > {T}_{c}\end{array}\end{array}$$

Secondly, antibody acquisition models describing the age and exposure dependent changes in antibody levels to a range of antigens were implemented using the methods described by Yman *et al*.^22^. Three variants of the model reflecting different assumptions on antigen immunogenicity and historical malaria exposure were tested.

Model 0: no age-dependent variation in geometric mean antibody level, with geometric mean antibody level in the 2003 cross-section equal to *α*_03_ and mean antibody level in 2013 equal to *α*_13_.

Model 1: geometric mean antibody level increases at a constant rate with age so that the geometric mean antibody level at age *a* is given by:

i $$\begin{array}{c}{A}_{03}(a)=\frac{{\alpha }_{03}}{\rho }(1-{e}^{-ra})\\ {A}_{13}(a)=\frac{{\alpha }_{13}}{\rho }(1-{e}^{-ra})\end{array}$$

Model 2: geometric mean antibody level increases constantly with age, but for the samples taken in the 2013 cross-section, we account for a sharp drop in transmission *T*_*c*_ years ago. The geometric mean antibody level at age *a* is given by:

ii $$\begin{array}{c}{A}_{03}(a)=\frac{{\alpha }_{03}}{r}(1-{e}^{-ra})\\ {A}_{13}(a)=\{\begin{array}{cc}\frac{{\alpha }_{13,c}}{r}(1-{e}^{-ra}) & a\le {T}_{c}\\ \frac{{\alpha }_{13,0}}{r}{e}^{-ra}({e}^{r{T}_{c}}-1)+\frac{{\alpha }_{13,c}}{r}(1-{e}^{-r(a-{T}_{c})}) & a > {T}_{c}\end{array}\end{array}$$

All models were fitted in a Bayesian framework using Markov Chain Monte Carlo (MCMC) methods with uninformative priors. A binomial likelihood was used for the sero-catalytic data where individuals are sero-negative or sero-positive. A log-Normal likelihood was used for the antibody acquisition model where there was a continuous distribution of antibody levels. The best fitting model was selected using the Deviance Information Criterion (DIC). Model selection based on DIC mitigates against the risk of selecting an overly parameterized model that the data cannot support.

### Data availability statement

Primary data related to antibody responses without personal identifiers and modeling of these data will be provided to readers upon request.

## Electronic supplementary material

Supplementary Data and Figure Legends

Supplementary Dataset 1
