# Supplementary material for: Sero-catalytic and Antibody Acquisition Models to Estimate Differing Malaria Transmission Intensities in Western Kenya
Source: Sci Rep. 2017 Dec 4;7:16821. doi: 10.1038/s41598-017-17084-9 (PMC5715086; doi:10.1038/s41598-017-17084-9)
Supplement: Supplementary file 1 — Supplementary Data and Figure Legends [file 41598_2017_17084_MOESM1_ESM.pdf]

Supplementary Data.

Sero-catalytic and Antibody Acquisition Models to Estimate Differing Malaria Transmission Intensities in Western Kenya

Grace E. Weber[1,2], Michael T. White[3], Anna Babakhanyan[1], Peter Odada Sumba[4], John Vulule[4], Dylan Ely[1], Chandy John[5], Evelina Angov[6], David Lanar[6], Sheetij Dutta[6], David L. Narum[7], Toshihiro Horii[8], Alan Cowman[9], James Beeson[10], Joseph Smith[11], James W. Kazura[1*], Arlene E. Dent[1,12]

[1]Center for Global Health and Diseases, Case Western Reserve University, Cleveland, OH, USA

[2]Department of Molecular Medicine, Cleveland Clinic Lerner College of Medicine, Case Western Reserve University, Cleveland, OH, USA

[3]Institute Pasteur, Paris, France

[4]Kenya Medical Research Institute, Kisumu, Kenya

[5]Department of Pediatrics, Riley Hospital, Indiana University, Indianapolis, IN, USA

[6]Malaria Vaccine Branch, Walter Reed Army Institute of Research, Silver Spring, MD, USA

[7]Laboratory of Malaria Immunology and Vaccinology, National Institutes of Allergy and Infectious Diseases, National Institutes of Health, Rockville, MD, USA

[8]Department of Molecular Protozoology, Research Institute for Microbial Diseases, Osaka University, Suita, Osaka, Japan

[9]Walter and Eliza Hall Institute of Medical Research, Parkville, VIC, Australia

[10]Burnet Institute, Melbourne, Australia

[11]Center for Infectious Disease Research, Seattle, WA, USA

[12]Department of Pediatrics, Rainbow Babies and Children's Hospital, Cleveland, OH, USA

Grace Weber and Michael White are shared first authors.

*Corresponding author email address jxk14@case.edu

Supplementary Data. Numerical expression of correlations among two-way comparison of antibodies to 28 *P. falciparum* antigens. These data correspond to the heat map presented in Figure 5.

Supplementary Figure S1. Breadth of antibody responses in 2003 cross section (solid black circles) and 2013 (open circles) by age groups. Each data point represents the number of antigens recognized by an individual. Median and interquartile ranges are shown for each age group. Differences in magnitudes by age groups are indicated (Kruskall Wallis test corrected for multiple comparisons). * $p<0.05$, ** $p<0.01$, *** $p<0.005$, **** $p<0.0001$.

Supplementary Table S1

Sero-catalytic and Antibody Acquisition Models to Estimate Differing Malaria Transmission Intensities in Western Kenya

Grace E. Weber[1,2], Michael T. White[3], Anna Babakhanyan[1], Peter Odada Sumba[4], John Vulule[4], Dylan Ely[1], Chandy John[5], Evelina Angov[6], David Lanar[6], Sheetij Dutta[6], David L. Narum[7], Toshihiro Horii[8], Alan Cowman[9], James Beeson[10], Joseph Smith[11], James W. Kazura[1*], Arlene E. Dent[1,12]

[1]Center for Global Health and Diseases, Case Western Reserve University, Cleveland, OH, USA

[2]Department of Molecular Medicine, Cleveland Clinic Lerner College of Medicine, Case Western Reserve University, Cleveland, OH, USA

[3]Institute Pasteur, Paris, France

[4]Kenya Medical Research Institute, Kisumu, Kenya

[5]Department of Pediatrics, Riley Hospital, Indiana University, Indianapolis, IN, USA

[6]Malaria Vaccine Branch, Walter Reed Army Institute of Research, Silver Spring, MD, USA

[7]Laboratory of Malaria Immunology and Vaccinology, National Institutes of Allergy and Infectious Diseases, National Institutes of Health, Rockville, MD, USA

[8]Department of Molecular Protozoology, Research Institute for Microbial Diseases, Osaka University, Suita, Osaka, Japan

[9]Walter and Eliza Hall Institute of Medical Research, Parkville, VIC, Australia

[10]Burnet Institute, Melbourne, Australia

[11]Center for Infectious Disease Research, Seattle, WA, USA

[12]Department of Pediatrics, Rainbow Babies and Children's Hospital, Cleveland, OH, USA

Grace Weber and Michael White are shared first authors.

*Corresponding author email address jxk14@case.edu

Protein	Concentration coupled to beads (μg/6.125 x 10^5 beads)	Source	Expression system	Reference
CSP	0.25	Evelina Angov	*E. coli*	[1]
LSA1	0.2	David Lanar	*E. coli*	[2]
Pf Celtos	1.0	Evelina Angov	*E. coli*	[3]
MSP1(42) 3D7	0.5	David Narum	*E. coli*	[4]
MSP1(42) FVO	0.6	David Narum	*E. coli*	[5]
MSP1(42) FUP	0.25	David Narum	*E. coli*	[6]
MSP2 Fc27	0.1	James Beeson	*E. coli*	[7]
MSP3	0.5	David Narum	*E. coli*	[8]
MSP6	0.3	James Beeson	*E. coli*	[9]
MSP7	0.5	James Beeson	*E. coli*	[10]
MSP DBL1	0.1	Alan Cowman	*E. coli*	[11]
MSP DBL2	0.075	Alan Cowman	*E. coli*	[11]
EBA140	0.1	Alan Cowman	*E. coli*	[12]
EBA175 W2Mef	0.05	Alan Cowman	*E. coli*	[13]
EBA175 3D7	0.08	Alan Cowman	*E. coli*	[14]
EBA181	0.1	Alan Cowman	*E. coli*	[15]
AMA1 3D7	0.3	David Narum	*Pichia pastoris*	[16]
AMA1 FVO	0.5	David Narum	*Pichia pastoris*	[17]
RH2	0.5	Alan Cowman	*E. coli*	[18]
RH4	0.8	Alan Cowman	*E. coli*	[19]
RH5	4.0	Alan Cowman (Genova)	*E. coli*	[20]
RIPR	8.0	Alan Cowman (Genova)	*E. coli*	[21]
SERA5	1.0	Toshihiro Horii	*E. coli*	[22]
DBLα2	0.5	Joseph Smith	*E. coli*	[23]
CIDRα1.1	0.5	Joseph Smith	*E. coli*	[23]
CIDRα1.4	0.5	Joseph Smith	*E. coli*	[23]
DBLβ12	0.5	Joseph Smith	*E. coli*	[23]
DBLγ6	0.5	Joseph Smith	*E. coli*	[23]

Supplementary Table 1. Sources of recombinant *Plasmodium falciparum* proteins, protein concentrations used for conjugation to Bioplex beads, expression vectors, and salient references describing the proteins.

References for Supplementary Table 1

1. Porter, M.D., et al., *Transgenic parasites stably expressing full-length Plasmodium falciparum circumsporozoite protein as a model for vaccine down-selection in mice using sterile protection as an endpoint.* Clin Vaccine Immunol, 2013. **20**(6): p. 803-10.
2. Hillier, C.J., et al., *Process development and analysis of liver-stage antigen 1, a preerythrocyte-stage protein-based vaccine for Plasmodium falciparum.* Infect Immun, 2005. **73**(4): p. 2109-15.
3. Bergmann-Leitner, E.S., et al., *Immunization with pre-erythrocytic antigen CelTOS from Plasmodium falciparum elicits cross-species protection against heterologous challenge with Plasmodium berghei.* PLoS One, 2010. **5**(8): p. e12294.
4. Angov, E., et al., *Development and pre-clinical analysis of a Plasmodium falciparum Merozoite Surface Protein-1(42) malaria vaccine.* Mol Biochem Parasitol, 2003. **128**(2): p. 195-204.
5. Darko, C.A., et al., *The clinical-grade 42-kilodalton fragment of merozoite surface protein 1 of Plasmodium falciparum strain FVO expressed in Escherichia coli protects Aotus nancymai against challenge with homologous erythrocytic-stage parasites.* Infect Immun, 2005. **73**(1): p. 287-97.
6. Angov, E., et al., *Heterologous protein expression is enhanced by harmonizing the codon usage frequencies of the target gene with those of the expression host.* PLoS One, 2008. **3**(5): p. e2189.
7. Reddy, S.B., et al., *High affinity antibodies to Plasmodium falciparum merozoite antigens are associated with protection from malaria.* PLoS One, 2012. **7**(2): p. e32242.
8. Tsai, C.W., et al., *Characterization of a protective Escherichia coli-expressed Plasmodium falciparum merozoite surface protein 3 indicates a non-linear, multi-domain structure.* Mol Biochem Parasitol, 2009. **164**(1): p. 45-56.
9. Pearce, J.A., et al., *Plasmodium falciparum merozoite surface protein 6 is a dimorphic antigen.* Infect Immun, 2004. **72**(4): p. 2321-8.
10. Richards, J.S., et al., *Identification and prioritization of merozoite antigens as targets of protective human immunity to Plasmodium falciparum malaria for vaccine and biomarker development.* J Immunol, 2013. **191**(2): p. 795-809.
11. Lin, C.S., et al., *The merozoite surface protein 1 complex is a platform for binding to human erythrocytes by Plasmodium falciparum.* J Biol Chem, 2014. **289**(37): p. 25655-69.
12. Thompson, J.K., et al., *A novel ligand from Plasmodium falciparum that binds to a sialic acid-containing receptor on the surface of human erythrocytes.* Mol Microbiol, 2001. **41**(1): p. 47-58.
13. Healer, J., et al., *Vaccination with conserved regions of erythrocyte-binding antigens induces neutralizing antibodies against multiple strains of Plasmodium falciparum.* PLoS One, 2013. **8**(9): p. e72504.
14. Reed, M.B., et al., *Targeted disruption of an erythrocyte binding antigen in Plasmodium falciparum is associated with a switch toward a sialic acid-independent pathway of invasion.* Proc Natl Acad Sci U S A, 2000. **97**(13): p. 7509-14.

15. Gilberger, T.W., et al., *A novel erythrocyte binding antigen-175 paralogue from Plasmodium falciparum defines a new trypsin-resistant receptor on human erythrocytes.* J Biol Chem, 2003. **278**(16): p. 14480-6.
16. Dutta, S., et al., *Purification, characterization, and immunogenicity of the refolded ectodomain of the Plasmodium falciparum apical membrane antigen 1 expressed in Escherichia coli.* Infect Immun, 2002. **70**(6): p. 3101-10.
17. Ellis, R.D., et al., *Phase 1 study in malaria naive adults of BSAM2/Alhydrogel(R)+CPG 7909, a blood stage vaccine against P. falciparum malaria.* PLoS One, 2012. **7**(10): p. e46094.
18. Triglia, T., et al., *Plasmodium falciparum merozoite invasion is inhibited by antibodies that target the PfRh2a and b binding domains.* PLoS Pathog, 2011. **7**(6): p. e1002075.
19. Tham, W.H., et al., *Antibodies to reticulocyte binding protein-like homologue 4 inhibit invasion of Plasmodium falciparum into human erythrocytes.* Infect Immun, 2009. **77**(6): p. 2427-35.
20. Reddy, K.S., et al., *Bacterially expressed full-length recombinant Plasmodium falciparum RH5 protein binds erythrocytes and elicits potent strain-transcending parasite-neutralizing antibodies.* Infect Immun, 2014. **82**(1): p. 152-64.
21. Reddy, K.S., et al., *Multiprotein complex between the GPI-anchored CyRPA with PfRH5 and PfRipr is crucial for Plasmodium falciparum erythrocyte invasion.* Proc Natl Acad Sci U S A, 2015. **112**(4): p. 1179-84.
22. Sugiyama, T., et al., *Production of recombinant SERA proteins of Plasmodium falciparum in Escherichia coli by using synthetic genes.* Vaccine, 1996. **14**(11): p. 1069-76.
23. Avril, M., et al., *A restricted subset of var genes mediates adherence of Plasmodium falciparum-infected erythrocytes to brain endothelial cells.* Proc Natl Acad Sci U S A, 2012. **109**(26): p. E1782-90.

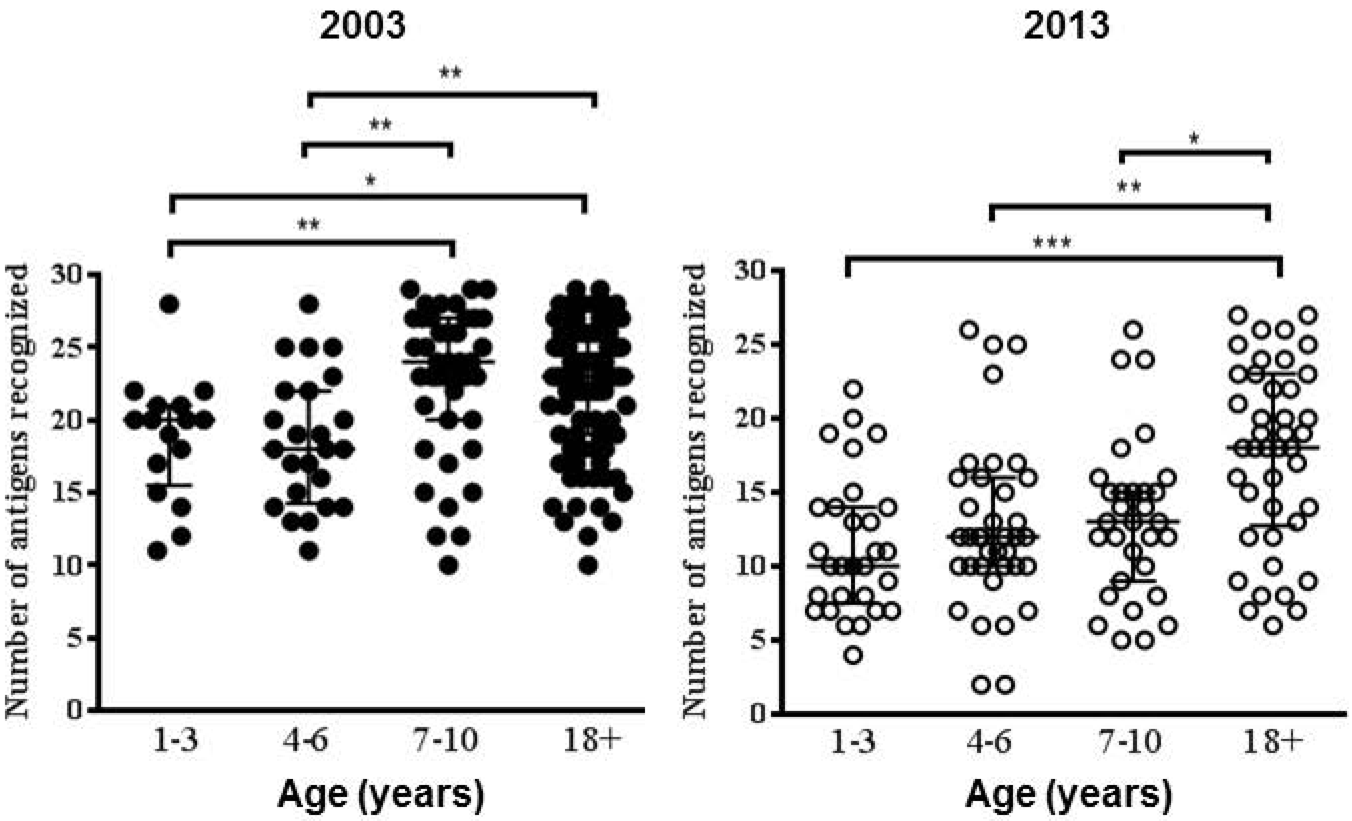
2003
2013
Number of antigens recognized
Age (years)
1-3
4-6
7-10
18+
0
5
10
15
20
25
30
*
**
***
